# Low-oil corn distillers dried grains with solubles can be fed to pigs up to 16.5% without compromising growth and pork quality

**DOI:** 10.5713/ab.24.0629

**Published:** 2024-11-06

**Authors:** Woon Sen Lee, Hyunwoong Jo, In Ho Kim, Beob Gyun Kim

**Affiliations:** 1 Department of Animal Science, Konkuk University, Seoul, Korea; 2 Monogastric Animal Feed Research Institute, Konkuk University, Seoul, Korea; 3 Department of Animal Resource and Science, Dankook University, Cheonan, Korea

**Keywords:** Carcass Characteristics, Growth Performance, Low-oil Distillers Dried Grains with Solubles, Pork Quality, Swine

## Abstract

**Objective:**

This study aimed to determine the maximum inclusion rate of low-oil corn distillers dried grains with solubles (DDGS) in swine diets with a focus on growth performance and pork quality.

**Methods:**

A total of 160 crossbred pigs with an initial body weight (BW) of 25.5 kg (standard deviation = 2.5) were randomly assigned to one of 5 dietary treatments in a randomized complete block design with BW and sex as blocking factors to obtain 8 replicates and 4 pigs per pen. The low-oil corn DDGS contained 26.3% crude protein, 24.5% neutral detergent fiber, and 3.7% ether extract. Five experimental diets based on the corn, soybean meal, and wheat were formulated to contain 0%, 10%, 20%, 30%, and 40% of the low-oil corn DDGS for each of 2 phases.

**Results:**

During the overall period, average daily gain, average daily feed intake, and gain-to-feed ratio linearly decreased (p<0.05) as the low-oil corn DDGS inclusion rate increased. A one-slope broken-line analysis showed that the maximum inclusion rate of low-oil corn DDGS in swine diets without compromising gain-to-feed ratio was 16.5% during the overall period. The carcass characteristics were not affected by the inclusion of low-oil corn DDGS up to 40%. The firmness of loin and belly linearly decreased (p<0.05) as the low-oil corn DDGS inclusion rate increased. As the inclusion rate of low-oil DDGS increased, the saturated fatty acid content in both loin and pork belly linearly decreased (p<0.001), whereas the unsaturated fatty acid content linearly increased (p<0.001). The iodine value of lard also showed a linear increase (p<0.001) with increasing the low-oil corn DDGS inclusion rate.

**Conclusion:**

The maximum inclusion rate of low-oil corn DDGS in growing-finishing swine diets without detrimental effects on growth performance and pork quality was 16.5%.

## INTRODUCTION

In the swine industry, one of the important goals is to efficiently produce pork at a low cost. As a result, extensive research has been conducted on economically viable alternative feed ingredients [1,2]. Corn distillers dried grains with solubles (DDGS), a byproduct from the dry-grind ethanol industry, have been widely used in swine diets as a cost-effective source of energy and nutrients [3]. However, the nutritional variability of corn DDGS due to different ethanol production process can cause nutritional imbalances [4–6]. The NRC [7] has classified corn DDGS into 3 categories based on their fat content: high-oil corn DDGS containing more than 10% oil, medium-oil corn DDGS containing between 6% and 9% oil, and low-oil corn DDGS containing less than 4% oil.

The variable nutrient content in corn DDGS, including unsaturated fatty acids (UFA) and fiber, can negatively affect pork quality and growth performance [8]. As a result, numerous studies have been conducted to determine the appropriate inclusion rates of corn DDGS in swine diets [9–11]. Up to 30% conventional corn DDGS containing approximately 10% ether extract can be used in diets for pigs [8], but the inclusion rate should be carefully decided depending on the fat concentration in corn DDGS [12]. Although a plethora of data for corn DDGS fed to pigs has been documented, most of the previous studies tested medium-oil and high-oil corn DDGS. However, the information on the effects of dietary low-oil corn DDGS on growing pigs is very limited in spite that this ingredient becomes more available for feed production [13]. To bridge this gap, the present study aimed to determine the appropriate inclusion rate of low-oil corn DDGS in pig diets with a focus on growth performance and pork quality.

## MATERIALS AND METHODS

The experimental protocols describing the management and care of animals were reviewed and approved by the Institutional Animal Care and Use Committee of Dankook University (DK-3-1705).

### Animals, diets, and experimental design

A total of 160 crossbred pigs ([Landrace×Yorkshire]×Duroc; 80 barrows and 80 gilts) with an initial body weight (BW) of 25.5 kg (standard deviation = 2.5) were allotted 5 dietary treatments in a randomized complete block design considering BW and sex as blocking factors using a spreadsheet-based program [14] to obtain 8 replicate pens per treatment and 4 pigs per pen. All animals were housed in concrete slotted floor pens (1.8 m×1.8 m) each equipped with a feeder and a nipple drinker and were allowed free access to feed and water during the entire experimental period. After feeding a common commercial diet for 13 days, experimental diets for phase 1 (days 0 to 42) and phase 2 (days 42 to 98) were provided to the pigs without reallotting the pigs. The low-oil corn DDGS used in this experiment was supplied by Poet Nutrition Inc. (Sioux Falls, SD, USA) and classified as low-oil corn DDGS (Table 1). The control diet was based on corn, soybean meal, and wheat in all phases (Table 2). Four additional experimental diets were formulated to contain varying levels of low-oil corn DDGS at 10%, 20%, 30%, and 40% replacing corn and soybean meal. All experimental diets maintained constant concentrations of energy, limiting amino acids (AA), calcium, and phosphorus to meet or exceed the nutrient requirement estimates suggested by the NRC [7].

### Growth performance, carcass characteristic, and pork quality

Individual BW of pigs was measured on day 0, 42, and 98 to calculate the average daily gain (ADG). The quantity of feed consumption was recorded on day 42 and 98 for the calculation of average daily feed intake (ADFI) and gain-to-feed ratio (G:F) for each pen.

At the end of the experiment, all pigs were slaughtered at a commercial slaughterhouse. Hot carcass weight was recorded after exsanguination and evisceration. The carcass backfat thickness was measured using a real-time ultrasound instrument (Piglot 105; SFK Technology, Herlev, Denmark). After chilling for 24 hours at 4°C, one pig was randomly selected from each replicate pen and a sample was obtained from the right loin between the 10th and 11th ribs. Additionally, the pork belly sample was separated from the pre-chilled carcass on the right side. After a 30-minute minimum bloom time, lightness (L*), redness (a*), and yellowness (b*) values at 3 locations on each sample surface were measured using a CR-410 Chroma Meter (Konica Minolta Sensing Americas, Inc., Ramsey, NJ, USA). Among the sensory test items (color, marbling, and firmness score), meat color and marbling were measured according to the detailed standards for livestock product grading specified in the Korean Ministry of Agriculture, Food and Rural Affairs Notice No. 2017–22 [15]. Firmness score was measured in accordance with NPPC [16]. At the same time, the pH values of each sample were measured at 2 different locations using a pH meter (Testo 205; Testo Pty Ltd, Croydon South, Australia), and the average was recorded. Subsequently, for water holding capacity (WHC) determination, 0.3 g of meat sample was placed on a filter paper of 125-mm diameter and pressed at 3,000 psi for 3 min. The moisture-exposed areas of the compressed sample were determined using a digitizing area-line sensor (MT-10S; M.T. Precision Co., Ltd., Tokyo, Japan). The ratio of water area to meat area was then calculated (a smaller ratio indicates increased WHC) and recorded. The measurements of longissimus muscle surface, cooking loss, and drip loss were based on the procedures described by Dang and Kim [17].

### Chemical analyses

The ingredients and experimental diets were finely ground to pass 1-mm screen before chemical analysis. The samples were analyzed for dry matter (method 930.15), crude protein (method 990.03), ether extract (method 920.39), ash (method 942.05), calcium (method 927.02), phosphorus (method 964.06), neutral detergent fiber (NDF; method 2002.04), and acid detergent fiber (method 973.18) as described in the AOAC [18]. Fatty acids (FA) were analyzed by gas chromatography using a Hewlett Packard HP-6890 gas chromatography equipped with a flame ionization detector and a capillary column HP-innowax (Agilent Technologies Inc., Santa Clara, CA, USA) following the procedure described by Zhao et al [19]. To determine the iodine value (IV), the procedure involved weighing 0.1 to 0.6 g of the extract into a 250 mL Erlenmeyer flask following the procedure suggested by Lo Fiego et al [20]. This was followed by dissolving it with 10 mL of chloroform and then adding 25 mL of Wijs reagent. After shaking until the solution turned transparent, it was left in a dark room at 25±5°C for 30 minutes. Subsequently, 20 mL of 10% potassium iodine solution was added, and 100 mL of distilled water was added to stop the reaction. Following this, 1% soluble starch solution was introduced, and titration was performed using 0.1 N-sodium thiosulfate solution until the solution turned colorless, indicating the endpoint.

### Statistical analysis

Data were analyzed by the MIXED procedure of SAS (SAS Institute Inc., Cary, NC, USA). The statistical model included dietary treatment as a fixed variable, and block as a random variable. Linear and quadratic effects of dietary treatment were analyzed using orthogonal polynomial contrasts. Least squares means were calculated for dietary treatments and the pen was considered the experimental unit. A broken-line analysis was conducted using the NLIN procedure to determine the maximum inclusion level of low-oil corn DDGS in swine diets as described by Robbins et al [21]. An alpha level of 0.05 was used to determine statistical significance.

## RESULTS

Pigs remained healthy and consumed their diets without apparent problems throughout the experimental period.

### Growth performance

**Figure 1:**
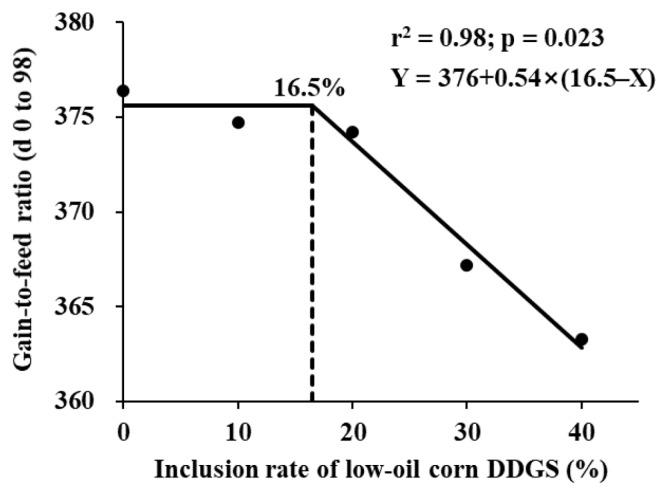
A one-slope broken-line analysis of the gain-to-feed ratio on days 0 to 98 according to inclusion rate of low-oil corn distillers dried grains with solubles (DDGS). Each data point represents least squares mean of 8 replicate pens with 4 pigs per pen. The broken-line model showed that maximum inclusion rate of low-oil corn DDGS was 16.5% (standard error = 2.91) based on the following equation: Y = 376+0.54×(16.5−X) where X is greater than 16.5 (r^2^ = 0.98; p = 0.023).

During phase 1 (days 0 to 42), ADG, G:F, and day 42 BW linearly decreased (p<0.05) with increasing dietary low-oil corn DDGS (Table 3). During phase 2 (days 42 to 98), ADG and final BW linearly decreased (p<0.05) with increasing dietary low-oil corn DDGS. During the overall period, increasing low-oil corn DDGS concentration led to a linear decrease (p<0.05) in ADG, ADFI, and G:F. The one-slope broken-line analysis showed that the maximum inclusion rate of low-oil corn DDGS in the diet was 16.5% based on G:F of the overall period (Figure 1).

### Carcass characteristics and pork quality

No effect of increasing dietary low-oil corn DDGS was observed on hot carcass weight, carcass yield, and backfat thickness (Table 4). The firmness score of loin linearly decreased (p = 0.022) with increasing dietary low-oil corn DDGS. Similarly, in the belly samples, a linear decrease in firmness score (p = 0.006) was observed with increasing dietary low-oil corn DDGS (Table 5). However, drip loss of belly on day 3 linearly increased (p = 0.029) with increasing dietary low-oil corn DDGS.

### Fatty acid profile and iodine value

In the loin samples, the concentrations of C16:0, C18:0, and total saturated fatty acids (SFA) linearly decreased (p<0.001) with increasing dietary low-oil corn DDGS (Table 6). Conversely, the concentration of total UFA increased linearly (p<0.001) with increasing dietary low-oil corn DDGS. The concentration of C18:1 decreased linearly (p = 0.005) whereas C18:2n−6 increased linearly (p<0.001) with increasing dietary low-oil corn DDGS. In the belly samples, the concentrations of total SFA and total UFA linearly decreased (p<0.001) and increased (p< 0.001) with increasing low-oil corn DDGS inclusion rate, respectively (Table 7). The IV in belly samples linearly increased (p<0.001) with increasing low-oil corn DDGS inclusion rate.

**Figure 2:**
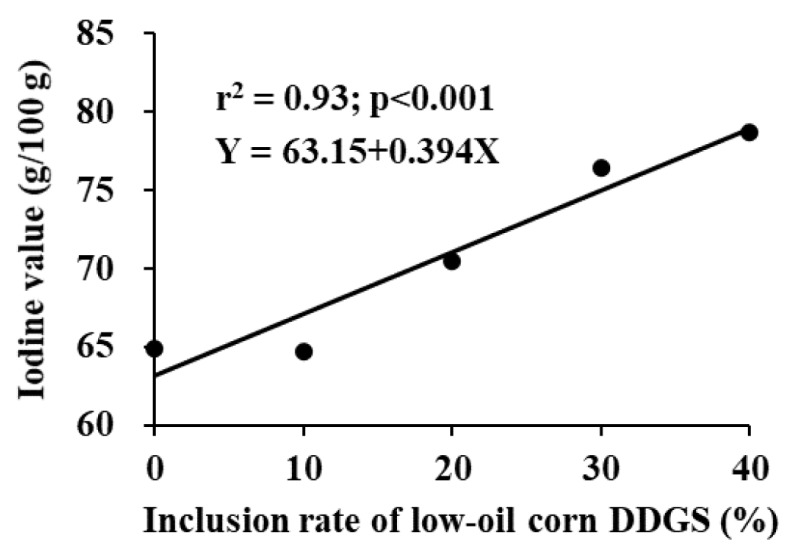
A regression analysis of the iodine value (IV) of lard according to inclusion rate of low-oil corn distillers dried grains with solubles (DDGS). Each data point represents least squares mean of 8 observations. The linear regression model of IV showed that inclusion rate of low-oil DDGS increased IV linearly based on following equation: Y = 63.15+0.394X (r^2^ = 0.93; p<0.001). The National Pork Producers Council set a standard for the maximum body fat IV at 70 g/100 g [16]. Based on the present results, the maximum allowable inclusion rate of low-oil DDGS in swine diet was approximately 17.3%.

The regression analysis showed that the inclusion rate of low-oil corn DDGS linearly increased (p<0.001; r^2^ = 0.93) the IV of belly (Figure 2). Based on the standard for the maximum body fat IV of 70 g/100 g [16], the maximum allowable inclusion rate of low-oil corn DDGS in swine diets was approximately 17.3%.

## DISCUSSION

### Growth performance

A number of studies have determined the effects of feeding corn DDGS on growth performance of pigs [9,22,23]. However, responses in ADG, ADFI, and G:F of pigs have been inconsistent when corn DDGS was included in diets based on a meta-analysis [24]. Jang et al [24] suggested that dietary corn DDGS reduced growth performance in 27% cases that are in agreement with the present study. However, dietary corn DDGS resulted in no performance changes in 65% cases and improved performance in 8% [24]. Jang et al [24] suggested that one of the primary reasons for the influence of dietary corn DDGS on the growth performance of pigs would be the inclusion rate of corn DDGS. In many studies, the inclusion of up to 30% corn DDGS in diets fed to growing-finishing pigs had no effect on growth performance of pigs [11,25,26]. However, the present study suggests that the maximum inclusion rate of low-oil corn DDGS should not exceed 16.5% based on G:F of overall period. One possible reason for the discrepancy could arise from differences in the quality of corn DDGS used. It is important to note that the AA digestibility varies among different DDGS sources [4,27]. Fastinger and Mahan [27] compared 5 types of corn DDGS and reported that standardized ileal digestibility of lysine ranged from 38.2% to 61.5%. Additionally, Stein et al [4] reported that standardized ileal digestibility of lysine of 10 sources of corn DDGS ranged from 43.9% to 63.0%. The standardized ileal digestibility of lysine in the corn DDGS used in the present work was assumed to be 57%. The variations in lysine digestibility can be attributed to the extent of heat treatment during the ethanol production process. Excessive heat exposure can cause adverse effects on nutrient stability, particularly in AA, due to the potential Maillard reactions occurring when ingredients containing AA and reducing sugars are subjected to heat and moisture [28]. As a result of the Maillard reactions, lysine becomes attached to reducing sugars, resulting in what is known as unreactive lysine. This form of lysine is biologically unavailable to pigs. Therefore, it is possible that the low-oil corn DDGS used in the present experiment underwent excessive heat treatment during the ethanol manufacturing process. Another possible reason for the discrepancy among the studies would be the different fiber concentrations in the corn DDGS sources. The NDF concentrations in corn DDGS were reported to vary from 20% to 33% (coefficient of variation = 19%) [8]. The NDF concentration of the low-oil corn DDGS used in this study was very comparable to that used by Wu et al [25] who reported no effect of dietary corn DDGS containing 6.2% ether extract on growth performance when used up to 30%. The inclusion of corn DDGS containing 40% NDF and 9.9% ether extract at 40% in grow-finishing pig diets did not cause any detrimental effects in the growth performance [29]. Overall, the influence of fiber concentrations in corn DDGS appears on the growth performance of pigs remains unclear.

### Carcass characteristics and pork quality

Despite the linear decrease in the final BW due to the dietary low-oil corn DDGS inclusion, the effect of dietary low-oil corn DDGS on hot carcass weight and carcass yield percentage was not statistically significant. This appears to be due to the larger variation in hot carcass weight compared with final weight based on the standard error of the means values. High-fiber diets have been known to increase viscera weight resulting in lower carcass yield [30,31] due to the thickened muscular walls of the gut over time to maintain motility and effectively process the fiber. If the fibers from corn DDGS affected viscera weight, carcass yield percentage would have been affected by the inclusion rate of corn DDGS, which was not observed in the present work. According to a review by Stein and Shurson [8], 8 studies showed reduced carcass yield percentage by feeding corn DDGS to pigs whereas 10 studies showed no changes. The inconsistency is likely due to the fiber concentrations in corn DDGS sources and the feeding period. However, the reason for this inconsistency remains unclear.

Pork quality involves complex compositional and physicochemical properties, including WHC, pork color, sensory quality, and processing yield. Many of these properties are strongly influenced by the postmortem changes in muscle pH [16]. The pH of pork decreases as glycogen in the muscles breaks down into lactic acid after slaughter [32]. The extent of this pH decline affects WHC, color, shelf-life, and cooking loss [33]. In the present study, the pH at 24-hour postmortem of pigs fed 0 to 40% low-oil corn DDGS was within the allowable range of 5.5 to 6.0 [34]. Additionally, the sensory responses including color and marbling were also within the acceptable range of 2 to 4 points [16]. Although the pH at 24-hour postmortem and sensory test results were not affected by dietary low-oil corn DDGS in the present work, the linear reduction of pork firmness due to the dietary low-oil corn DDGS was observed. Similarly, Whitney et al [9] reported that increasing dietary corn DDGS up to 30% resulted in a linear decrease in pork belly firmness. The changes in pork firmness are likely attributed to the high contents of UFA in corn DDGS [22] despite the relatively low ether extract concentration in the corn DDGS used in the present experiment.

### Fatty acid profile and iodine value

The FA profile in animal tissues is primarily influenced by the synthesis of FA in animals and fat deposition from diets. As de novo synthesis of FA is prohibited by the sufficient supply of dietary FA in pigs, the FA contents in pig adipose tissues are largely affected by dietary FA [35]. The large quantity of C18:2 in pork fat indicates that a large portion of dietary fat contents are retained in pig adipose tissue as C18:2 is absorbed from the diets rather than synthesized by pigs [36]. In the present study, the concentration of dietary C18:2 increased from 1.7% to 2.3% as the dietary low-oil corn DDGS inclusion rate increased from 0% to 40%, which resulted in a linear increase in the C18:2n−6 content of body fat in pigs. Because UFA are more susceptible to oxidation and consist of a more flexible structure, softer fats are less capable of maintaining structural integrity. Consequently, the increased concentrations of UFA due to the dietary low-oil corn DDGS would have led to greater water exudation from pork belly measured as drip loss in the present study. When pigs consume diets rich in UFA, the metabolic pathways that process these FA become more active [37,38]. This increased activity can suppress the synthesis and accumulation of SFA, as the metabolic machinery prioritizes the processing of the more abundant UFA. A previous study reported that increasing dietary corn DDGS (0%, 10%, 20%, and 30%) led to a linear decrease in SFA content in pork belly, whereas the concentration of C18:2 linearly increased, consistent with our experiment [11].

The IV of pork fat provides an overall estimate of FA unsaturation, which serves as an indicator of firmness of fat [12]. Due to concerns regarding soft pork fat, some pork processors have established a maximum acceptance threshold for carcass fat IV, usually below 70 g/100 g [16]. In a previous study, even when a dietary inclusion rate of corn DDGS was increased up to 20%, the IV did not exceed 70 [11], which is similar to our experiment. However, another study found that the IV exceeded 70 for diets containing 20% corn DDGS [9]. Xu et al [11] did not include additional oil whereas Whitney et al [9] used soybean oil, rich in UFA, which could have influenced the FA composition of pork, indicating that the variable responses in pork fat IV in pigs fed corn DDGS are likely due to the additional oil source. In the present study, animal fat was used to maintain consistent energy values among experimental diets.

## CONCLUSION

Growth performance decreased when low-oil corn DDGS were included in diets at greater than 16.5% for growing-finishing pigs. The maximum inclusion rate of dietary low-oil corn DDGS was 17.3% to achieve pork fat IV of less than 70 g per 100 g. Therefore, feeding a diet containing up to 16.5% low-oil corn DDGS to grower-finisher pigs had no major adverse effects on growth performance and pork quality.

## Figures and Tables

**Table 1:** Analyzed nutrient composition of feed ingredients (as-fed basis)

Item (%)	Corn	Soybean meal	Wheat	Low-oil corn DDGS
Dry matter	85.74	87.78	88.30	88.50
Crude protein	6.85	45.80	12.13	26.30
Ether extract	3.69	1.78	1.44	3.72
Ash	1.13	6.14	1.50	5.50
Neutral detergent fiber	7.11	7.75	10.21	24.52
Acid detergent fiber	2.02	4.36	3.06	8.14
Calcium	0.06	0.41	0.08	0.08
Phosphorus	0.25	0.68	0.31	1.03

DDGS, distillers dried grains with solubles.

**Table 2:** Ingredient and chemical compositions of experimental diets for growing and finishing pigs containing low-oil corn distillers dried grains with solubles (DDGS; as-fed basis)

Item	Low-oil corn DDGS in phase 1 (days 0 to 42)					Low-oil corn DDGS in phase 2 (days 42 to 98)				
	
	0%	10%	20%	30%	40%	0%	10%	20%	30%	40%
Ingredient (%)										
Ground corn	62.11	57.04	51.98	46.92	41.85	65.70	60.64	55.57	50.51	45.34
Soybean meal (46% CP)	23.50	18.28	13.06	7.85	2.63	20.98	15.76	10.54	5.33	-
Wheat	10.00	10.00	10.00	10.00	10.00	10.00	10.00	10.00	10.00	10.00
Low-oil corn DDGS	-	10.00	20.00	30.00	40.00	-	10.00	20.00	30.00	40.00
Animal fat	1.40	1.62	1.84	2.06	2.28	1.12	1.34	1.56	1.78	2.08
l-Lys·HCl (79%)	0.38	0.49	0.60	0.72	0.83	0.17	0.28	0.40	0.51	0.63
dl-Met (99%)	0.05	0.04	0.04	0.03	0.02	0.03	0.02	0.01	0.01	0.00
l-Thr (99%)	0.10	0.12	0.15	0.17	0.20	0.02	0.04	0.07	0.09	0.12
l-Trp (99%)	0.01	0.03	0.05	0.06	0.08	0.00	0.02	0.04	0.06	0.07
Dicalcium phosphate	1.01	0.76	0.51	0.25	-	0.75	0.50	0.25	-	-
Ground limestone	0.85	1.02	1.18	1.35	1.51	0.73	0.90	1.06	1.22	1.26
Vitamin-mineral premix^1)^	0.30	0.30	0.30	0.30	0.30	0.30	0.30	0.30	0.30	0.30
Sodium chloride	0.30	0.30	0.30	0.30	0.30	0.20	0.20	0.20	0.20	0.20
Calculated composition (%)										
ME (kcal/kg)	3,330	3,330	3,330	3,330	3,330	3,330	3,330	3,330	3,330	3,330
CP	17.18	17.24	17.30	17.36	17.42	15.99	16.05	16.11	16.17	16.17
SID Lys	1.02	1.02	1.02	1.02	1.02	0.80	0.80	0.80	0.80	0.80
SID Met	0.30	0.30	0.30	0.30	0.30	0.26	0.26	0.26	0.26	0.26
SID Thr	0.62	0.62	0.62	0.62	0.62	0.51	0.51	0.51	0.51	0.51
SID Trp	0.17	0.17	0.18	0.17	0.17	0.15	0.15	0.15	0.15	0.15
Ether extract	4.03	4.44	4.84	5.25	5.66	3.81	4.21	4.62	5.03	5.51
Calcium	0.69	0.69	0.69	0.69	0.69	0.58	0.58	0.58	0.58	0.58
STTD phosphorus	0.33	0.33	0.33	0.33	0.33	0.28	0.28	0.28	0.28	0.32
Analyzed composition (%)										
CP	17.49	17.12	16.83	16.63	16.65	14.93	15.49	16.56	15.28	15.84
Ether extract	4.03	3.94	5.15	5.24	5.83	4.01	4.05	4.52	5.02	5.54
Neutral detergent fiber	7.36	8.99	10.82	12.30	13.54	10.69	10.81	11.44	13.24	15.26
Calcium	0.60	0.69	0.71	0.63	0.59	0.68	0.63	0.64	0.57	0.57
Total phosphorus	0.58	0.57	0.58	0.56	0.57	0.48	0.48	0.47	0.48	0.50
C18:2	1.63	1.59	2.07	2.08	2.32	1.71	1.73	1.92	2.16	2.36

1) Provided the following quantities per kg of complete diet: vitamin A, 7,000 IU; vitamin D_3_, 1,500 IU; vitamin E, 40 mg; vitamin K_3_, 1.5 mg; thiamin, 2 mg; riboflavin, 4 mg; pyridoxine, 2.7 mg; vitamin B12, 0.02 mg; pantothenic acid, 29.4 mg; folic acid, 0.58 mg; niacin, 20 mg; biotin, 0.15 mg; Co, 0.5 mg as cobalt sulfate; Cu, 50 mg as copper sulfate; Fe, 100 mg as iron sulfate; I, 2 mg as calcium iodate; Mg, 5 mg as magnesium sulfate; Mn, 30 mg as manganese sulfate; Se, 0.25 mg as sodium selenite; Zn, 40 mg as zinc.

CP, crude protein; Lys, lysine; Met, methionine; Thr, threonine; Trp, tryptophane; ME, metabolizable energy; SID, standardized ileal digestible; STTD, standardized total tract digestible.

**Table 3:** Effects of dietary low-oil corn distillers dried grains with solubles (DDGS) on growth performance of growing and finishing pigs^1)^

Item	Low-oil corn DDGS					SEM	p-value	
	
	0%	10%	20%	30%	40%		Linear	Quadratic
Days 0 to 42								
Initial body weight (kg)	32.9	33.0	33.1	33.0	33.1	0.83	0.231	0.641
Average daily gain (g/d)	710	704	694	684	674	10.9	0.012	0.818
Average daily feed intake (g/d)	1,708	1,705	1,682	1,695	1,668	20.8	0.175	0.894
Gain:feed (g/kg)	415	413	413	403	404	3.9	0.014	0.783
Final body weight (kg)	62.7	62.5	62.2	61.7	61.4	1.06	0.020	0.761
Days 42 to 98								
Average daily gain (g/d)	888	887	864	854	838	15.6	0.011	0.783
Average daily feed intake (g/d)	2,604	2,607	2,548	2,559	2,558	22.5	0.051	0.466
Gain:feed (g/kg)	341	340	339	334	328	5.1	0.050	0.455
Final body weight (kg)	112.5	112.2	110.6	109.5	108.3	1.58	0.011	0.765
Days 0 to 98								
Average daily gain (g/d)	812	808	791	781	768	12.3	0.006	0.775
Average daily feed intake (g/d)	2,156	2,156	2,115	2,127	2,113	17.9	0.044	0.705
Gain:feed (g/kg)	376	375	374	367	363	4.0	0.011	0.467

1) Each least squares mean represents 8 replicate pens with 4 pigs per pen.

SEM, standard error of the mean.

**Table 4:** Effects of dietary low-oil corn distillers dried grains with solubles (DDGS) on carcass characteristics and the quality of pork loin^1)^

Item	Low-oil corn DDGS					SEM	p-value	
	
	0%	10%	20%	30%	40%		Linear	Quadratic
Carcass characteristics^2)^								
Hot carcass weight (kg)	91.6	91.2	90.0	89.4	88.5	1.80	0.104	0.939
Carcass yield (%)	81.4	81.3	81.3	81.6	81.6	0.78	0.753	0.823
Backfat thickness (mm)	19.3	19.1	19.1	18.8	18.5	0.79	0.360	0.835
Loin muscle area (cm^2^)	61.3	61.0	60.7	60.2	59.6	1.24	0.253	0.829
Quality of pork loin^3)^								
Ultimate pH^4)^	5.57	5.54	5.52	5.55	5.57	0.054	0.925	0.531
Water holding capacity (%)	37.0	37.5	34.7	34.8	32.6	3.54	0.318	0.854
Meat color^5)^								
Minolta L*	51.7	50.7	51.6	50.4	51.3	1.59	0.806	0.781
Minolta a*	14.2	14.9	15.0	14.8	14.2	0.59	0.932	0.210
Minolta b*	3.85	3.65	3.77	3.56	3.65	0.347	0.648	0.852
Cooking loss (%)	26.0	26.6	27.6	28.2	29.3	2.08	0.199	0.928
Sensory test								
Color	3.19	3.24	3.13	3.11	3.22	0.115	0.836	0.522
Marbling	2.71	2.77	2.72	2.74	2.72	0.054	0.983	0.654
Firmness	3.13	3.08	2.88	2.94	2.85	0.097	0.022	0.625
Drip loss (%)								
Day 1	5.07	4.85	4.91	4.75	4.73	0.727	0.735	0.948
Day 3	11.9	12.1	11.9	12.0	11.5	1.00	0.730	0.731

1) Dietary treatments were fed from days 0 to 42 for phase 1 and days 42 to 98 for phase 2.

2) Each least squares mean represents 8 replicate pens with 4 pigs per pen.

3) Each least squares mean represents 8 observations.

4) After chilling for 24 hours at 4°C, the pH values of each sample were measured at 2 different locations using a pH meter (Testo 205; Testo Pty Ltd, Croydon South, Australia), and the average was recorded.

5) After a 30-minute minimum bloom time, lightness (L*), redness (a*), and yellowness (b*) values at 3 locations on each sample surface of pork loin were measured.

SEM, standard error of the mean.

**Table 5:** Effects of dietary low-oil corn distillers dried grains with solubles (DDGS) on the quality of pork belly^1),2)^

Item	Low-oil corn DDGS					SEM	p-value	
	
	0%	10%	20%	30%	40%		Linear	Quadratic
Ultimate pH^3)^	5.47	5.57	5.58	5.48	5.54	0.049	0.721	0.347
Water holding capacity (%)	35.9	35.8	34.6	33.4	32.9	2.55	0.304	0.944
Meat color^4)^								
Minolta L*	52.8	53.6	52.3	53.6	53.5	1.44	0.734	0.873
Minolta a*	13.8	13.8	14.0	13.5	13.2	0.64	0.430	0.496
Minolta b*	3.97	4.01	4.28	3.89	4.01	0.319	0.965	0.676
Cooking loss (%)	29.9	29.8	28.9	28.1	27.1	1.98	0.224	0.826
Sensory test								
Color	3.28	3.35	3.21	3.33	3.28	0.118	0.958	0.914
Marbling	2.75	2.72	2.71	2.69	2.75	0.056	0.866	0.374
Firmness	3.16	3.05	2.99	2.86	2.83	0.091	0.006	0.783
Drip loss (%)								
Day 1	4.58	4.75	4.58	4.87	5.14	0.503	0.442	0.720
Day 3	10.6	10.7	11.8	12.2	12.4	0.70	0.029	0.821

1) Dietary treatments were fed from days 0 to 42 for phase 1 and days 42 to 98 for phase 2.

2) Each least squares mean represents 8 observations.

3) After chilling for 24 hours at 4°C, the pH values of each sample were measured at 2 different locations using a pH meter (Testo 205; Testo Pty Ltd, Croydon South, Australia), and the average was recorded.

4) After a 30-minute minimum bloom time, lightness (L*), redness (a*), and yellowness (b*) values at 3 locations on each sample surface of pork belly were measured.

SEM, standard error of the mean.

**Table 6:** Effects of dietary low-oil corn distillers dried grains with solubles (DDGS) on fatty acids profile of pork loin^1),2)^

Item	Low-oil corn DDGS					SEM	p-value	
	
	0%	10%	20%	30%	40%		Linear	Quadratic
Fatty acids (%)								
C8:0	0.02	0.01	0.01	0.01	0.01	0.004	0.027	0.258
C10:0	0.07	0.07	0.08	0.07	0.06	0.005	0.220	0.056
C12:0	0.13	0.14	0.14	0.15	0.12	0.013	0.581	0.151
C14:0	1.42	1.36	1.38	1.36	1.19	0.082	0.089	0.432
C15:0	0.10	0.09	0.11	0.12	0.10	0.008	0.319	0.096
C16:0	23.47	23.11	21.11	20.58	20.35	0.403	<0.001	0.219
C17:0	0.58	0.56	0.55	0.58	0.57	0.055	0.988	0.813
C18:0	12.32	12.47	10.20	10.40	9.61	0.477	<0.001	0.696
C20:0	0.16	0.13	0.13	0.14	0.15	0.016	0.783	0.079
C14:1	0.08	0.03	0.04	0.10	0.10	0.036	0.332	0.323
C15:1	0.10	0.04	0.09	0.12	0.11	0.026	0.188	0.457
C16:1	3.05	2.64	2.78	2.68	2.69	0.133	0.101	0.202
C17:1	0.46	0.49	0.46	0.37	0.39	0.040	0.049	0.593
C18:1	40.61	39.59	39.25	38.18	38.33	0.688	0.005	0.488
C18:2n−6	14.50	16.12	20.14	21.72	23.20	0.742	<0.001	0.297
C18:3n−3	0.68	0.70	0.75	0.70	0.64	0.038	0.582	0.076
C18:3n−5	0.08	0.11	0.13	0.11	0.08	0.011	0.628	<0.001
C18:4n−3	0.28	0.18	0.22	0.21	0.14	0.029	0.003	0.804
C20:1n−9	0.77	0.87	0.91	0.75	0.72	0.050	0.172	0.017
Total SFA (%)	38.26	37.94	33.72	33.41	32.15	0.829	<0.001	0.417
Total UFA (%)	60.55	60.75	64.77	64.92	66.13	1.255	<0.001	0.475
UFA:SFA	1.61	1.61	1.92	1.95	1.95	0.075	<0.001	0.247

1) Dietary treatments were fed from day 0 to 42 for phase 1 and day 42 to 98 for phase 2.

2) Each least squares mean represents 8 observations.

SEM, standard error of the mean; SFA, saturated fatty acid; UFA, unsaturated fatty acid.

**Table 7:** Effects of dietary low-oil corn distillers dried grains with solubles (DDGS) on fatty acids profile of pork belly^1),2)^

Item	Low-oil corn DDGS					SEM	p-value	
	
	0%	10%	20%	30%	40%		Linear	Quadratic
Fatty acids (%)								
C8:0	0.01	0.01	0.01	0.01	0.06	0.009	0.004	0.009
C10:0	0.10	0.09	0.09	0.07	0.06	0.007	<0.001	0.721
C12:0	0.16	0.17	0.15	0.15	0.10	0.008	<0.001	0.003
C14:0	1.68	1.68	1.51	1.38	1.04	0.058	<0.001	0.006
C15:0	0.03	0.03	0.09	0.11	0.12	0.012	<0.001	0.823
C16:0	24.35	24.30	22.15	21.12	19.89	0.351	<0.001	0.293
C17:0	0.59	0.51	0.55	0.54	0.53	0.048	0.492	0.570
C18:0	12.30	12.10	9.64	10.30	9.62	0.449	<0.001	0.190
C20:0	0.13	0.13	0.11	0.11	0.13	0.007	0.294	0.086
C14:1	0.01	0.01	0.03	0.03	0.03	0.004	<0.001	0.461
C15:1	0.11	0.09	0.06	0.03	0.03	0.011	<0.001	0.328
C16:1	3.11	2.85	3.02	2.61	2.74	0.109	0.007	0.607
C17:1	0.44	0.38	0.45	0.43	0.43	0.033	0.824	0.835
C18:1	39.99	40.20	40.27	38.82	38.91	0.820	0.130	0.520
C18:2n−6	14.01	14.56	18.88	21.16	22.63	0.800	<0.001	0.943
C18:3n−3	0.71	0.64	0.69	0.67	0.63	0.037	0.177	0.889
C18:3n−5	0.08	0.07	0.09	0.11	0.08	0.012	0.507	0.471
C18:4n−3	0.11	0.09	0.12	0.14	0.31	0.033	<0.001	0.006
C20:1n−9	0.73	0.76	0.79	0.79	0.84	0.038	0.048	0.936
Total SFA (%)	39.36	39.01	34.28	33.75	31.53	0.701	<0.001	0.841
Total UFA (%)	59.31	59.65	64.39	64.79	66.63	0.670	<0.001	0.525
UFA:SFA	1.52	1.53	1.88	1.93	2.12	0.055	<0.001	0.728
Iodine value (g/100 g)	64.9	64.7	70.5	76.4	78.7	1.26	<0.001	0.266

1) Dietary treatments were fed from days 0 to 42 for phase 1 and days 42 to 98 for phase 2.

2) Each least squares mean represents 8 observations.

SEM, standard error of the mean; SFA, saturated fatty acid; UFA, unsaturated fatty acid.
